# Supported biopsychosocial self-management for back-related leg pain: a randomized feasibility study integrating a whole person perspective

**DOI:** 10.1186/s12998-025-00570-7

**Published:** 2025-02-05

**Authors:** Brent Leininger, Roni Evans, Carol M. Greco, Linda Hanson, Craig Schulz, Michael Schneider, John Connett, Francis Keefe, Ronald M. Glick, Gert Bronfort

**Affiliations:** 1 Integrative Health and Wellbeing Research Program, Earl E. Bakken Center for Spirituality and Healing, University of Minnesota, Mayo Memorial Building C504, 420 Delaware Street, Minneapolis, MN 55414 USA; 2 School of Medicine, Department of Psychiatry, University of Pittsburgh, 580 S. Aiken Avenue, Suite 310, Pittsburgh, PA 15232 USA; 3 Doctor of Chiropractic Program, School of Health and Rehabilitation Sciences, University of Pittsburgh, Bridgeside Point 1, 100 Technology Drive, Suite 500, Pittsburgh, PA 15219 USA; 4 School of Public Health, Division of Biostatistics, University of Minnesota, 717 Delaware Street SE, 2Nd Floor, Minneapolis, MN 5455 USA; 5 Pain Prevention and Treatment Research Program, Department of Psychiatry and Behavioral Sciences, Duke Medical Center, Duke University School of Medicine, Box 3159, Durham, NC 27705 USA; 6 School of Medicine, Departments of Psychiatry and Physical Medicine and Rehabilitation, University of Pittsburgh, 580 S. Aiken Avenue, Suite 310, Pittsburgh, PA 15232 USA

**Keywords:** Back-related leg pain, Self-management, Feasibility

## Abstract

**Background:**

There is limited high-quality research examining conservative treatments for back-related leg pain (BRLP). This feasibility study was done in preparation for a full-scale trial comparing a whole-person supported self-management intervention to medical care for chronic BRLP.

**Methods:**

Participants were randomized to 12 weeks of individualized supported self-management delivered by physical therapists and chiropractors or medical care consisting of guideline-based pharmacologic care. Supported self-management was based on a behavioral model that used a whole person approach to enhance participants capabilities, opportunities, and motivations to engage in self-care. It combined BRLP education with psychosocial strategies (e.g., relaxed breathing, progressive muscle relaxation, guided imagery, communication skills) and physical modalities such as exercise and spinal manipulation therapy. Providers were trained to address participants’ individualized needs and use behavior change and motivational communication techniques to develop a therapeutic alliance to facilitate self-management. Feasibility was assessed using pre-specified targets for recruitment and enrollment, intervention delivery, and data collection over the six-month study period. In addition, areas for potential refinement and optimization of processes and protocols for the full-scale trial were assessed.

**Results:**

We met or exceeded nearly all feasibility targets. Forty-two participants were enrolled over a six-month period in 2022 and very few individuals declined participation due to preferences for one treatment. All but one participant received treatment and 95% of participants attended the minimum number of visits (self-management = 6, medical care = 2). At 12 weeks, 95% of participants in the self-management group reported engaging in self-management practices learned in the program and 77% of medical care participants reported taking medications as prescribed. Satisfaction with the self-management intervention was high with 85% of participants reporting satisfaction with the program overall. Self-management intervention providers delivered all required activities at 72% of visits. Providers also noted some challenges navigating the shared decision-making process and deciding what self-management tools to prioritize. Over the six-month study period, completion rates were 91% for monthly surveys and 86% for weekly surveys.

**Conclusion:**

We were able to demonstrate that a full-scale randomized trial comparing a whole-person supported self-management intervention to medical care for chronic BRLP is feasible and identified important areas for optimization.

## Background

Back related leg pain (BRLP), also referred to as sciatica, is one of the most burdensome variations of the prevalent LBP conditions impacting 30 to 60% of those with LBP [1, 2]. BRLP is characterized by radiating pain originating from the lumbar spine and traveling into the proximal or distal lower extremity with or without neurologic signs [3, 4]. BRLP is a complex condition influenced by a web of interrelated physical, psychological, and social factors. And is associated with greater pain severity, disability, depression, anxiety, and social interference than LBP alone [1, 2, 5]. Those with BRLP are also more likely to miss work or be unemployed and use more healthcare including repeat general practitioner visits, physical therapy referrals, and hospitalizations than those with LBP alone [6]. In the U.S., BRLP has annual healthcare costs that are 2.5 times higher compared to LBP alone [7]. Further, these more complicated BRLP cases are more likely to be prescribed opioids, undergo diagnostic imaging, visit an ER, become hospitalized, and receive spinal surgery, [7] all of which are associated with increased risks and costs.

A cause-effect relationship between imaging findings and BRLP can rarely be established with certainty, as these findings have limited impact on clinical outcomes and are common in asymptomatic individuals [8–10]. Given this, it’s critical to shift focus away from treatments directed towards pathoanatomical findings to whole person approaches addressing important biopsychosocial (BPS) factors (e.g., stress, lack of social support). While the BPS model has been promoted for the past several decades [11, 12] most treatment approaches still fail to address the comprehensive range of interwoven factors implicated in BRLP and LBP conditions [13].

While evidence-based national and international guidelines advocate several complementary modalities as alternatives to drugs and other invasive treatments for chronic LBP conditions, there are no specific recommendations for the management of BRLP due to the limited amount of high-quality research [14, 15]. There is emerging evidence supporting the use of conservative treatments including manual therapies and exercise relative to home exercise [3] and usual care [16]. However, treatment effects are modest, and studies have not addressed BRLP from a BPS perspective.

Importantly, BRLP like most chronic conditions requires ongoing self-management, where patients actively participate and take responsibility for managing their health [17–19]. While patients often recognize this need, they often face BPS related capability, opportunity and motivational barriers, making it difficult to initiate and maintain self-management successfully without provider support (e.g., education, skill training, enablement, persuasion) [20, 21]. It has been estimated almost half of patients don’t engage in self-management and two thirds don’t adhere to prescribed home exercise, increasing the risk for poor outcomes and the use of more invasive treatments [22]. Indeed, self-management is a complex human behavior, requiring attentiveness to patients’ BPS needs and risk factors to increase engagement [23]. Important components of self-management interventions not only include specific tools (e.g. physical exercises, mind–body strategies like relaxed breathing) but also how a provider interacts and supports a patient. This includes taking a person-centered approach that emphasizes a productive therapeutic or working alliance characterized by shared decision making and collaboration regarding agreed-upon goals and how to achieve them, as well as a mutual inter-personal bond involving trust, acceptance and confidence [24, 25]. While there is growing evidence that interventions that embrace behavior change can improve patient adherence, [26, 27] their systematic application is underutilized in musculoskeletal pain research [28, 29].

Physical therapists (PTs) and chiropractors (DCs) are the most common providers of conservative treatment for LBP in the US [30, 31]. This makes them optimally positioned for integrating patient-centered psychosocial strategies to complement biophysical approaches [32, 33], and play a critical role in the frontline management of BRLP [34, 35]. Over the past decade there have been promising shifts in both the PT and DC fields to integrate more psychosocial strategies to better support patient self-management [25, 32, 33, 36–41]. However, effectively supporting behavior change and implementing the BPS model to support a whole person approach to complex conditions can be challenging to implement and effectively study due to clinicians’ lack of training and skills [42–45].

Well-designed feasibility studies provide an opportunity to systematically develop and assess new approaches to interventions in a manner that will increase the likelihood of successful implementation in future trials, and subsequent clinical practice. Key questions center around the ability to recruit and retain participants, deliver interventions with fidelity and satisfactory participant engagement which are common methodological shortcomings in many studies, including clinical trials for chronic pain [46–48].

### Objectives

This feasibility study was performed in anticipation of a full-scale randomized trial comparing a whole-person supported self-management intervention to medical care for chronic back-related leg pain. The objective was to assess feasibility using pre-specified targets for recruitment and enrollment (e.g., #’s screened and enrolled/month), intervention delivery (e.g., #’s attending minimum visits and satisfied with intervention) and data collection (e.g., #’s completing weekly and monthly surveys).

## Methods

### Study design and setting

The study was conducted at an outpatient research clinic at the University of Minnesota (UMN) from December 2021 through February 2023. The study was funded by the National Institute of Health’s National Center for Complementary and Integrative Health (R34AT011209) and registered at clinicaltrials.gov (NCT05022121). The pilot study used a parallel group randomized design. The RE-AIM framework was used to inform potential refinement of the future full-scale trial, with a focus on reach and implementation [49]. The UMN’s Institutional Review Board approved the study (STUDY00013265) and all participants provided electronic consent for screening and study enrollment. An independent monitoring committee reviewed and monitored the study.

### Participants

Participants were 18 years of age or older with chronic (12 weeks or longer) back-related leg pain consistent with Quebec Task Force (QTF) categories 2–4. This includes radiating pain to the proximal or distal extremity with or without neurological signs (decreased sensation, strength, or reflexes in the lower extremity). An average back-related leg pain severity of 3 or higher in the past week (0 to 10 scale) was required at all screening visits and participants had to be able to communicate in English.

Participants were excluded for the following reasons: central spinal stenosis (QTF category 7); specific, non-mechanical cause of back-related leg pain (e.g., infection, cancer); contraindications to study interventions (e.g., spinal fractures (QTF category 5)); inflammatory conditions of the lumbar spine (QTF category 11); surgical fusion of the lumbar spine; progressive neurological deficits; cauda equina syndrome; pregnant or nursing mothers; severe unmanaged comorbid conditions (e.g., substance abuse, stage 3 hypertension); or receiving ongoing back-related leg pain care from another provider. Individuals with arthritis in the lower extremity were excluded if the arthritic pain could not be clearly distinguished from BRLP.

### Recruitment

Individuals were recruited from the general population using a variety of approaches including direct post-card mailings and social media advertisements (e.g. Facebook). Other recruitment methods included electronic and print postings through UMN affiliated newsletters, websites, social media pages, and clinics; registration on ResearchMatch and StudyFinder; and sharing information about the study with community-based partner organizations and other ongoing clinical studies for pain at the UMN.

### Screening

Participants completed 4 screening stages prior to enrollment. After completing a web-based survey to determine initial eligibility, they were called by a physical therapist or chiropractor who conducted a more detailed assessment of inclusion and exclusion criteria. Eligible participants then attended an in-person baseline evaluation that included informed consent, a health history, and a physical examination by a licensed physical therapist or chiropractor. The physical examination was focused on the back and lower extremities and included assessments of posture, gait, range of motion, palpation for spinal mobility and tenderness, and orthopedic and neurological tests (e.g., straight leg raise, lower extremity sensation, reflexes, and muscle strength). The physical exam was performed to confirm eligibility criteria by ensuring leg symptoms were back-related and that no contraindications to study interventions existed (e.g., progressive neurological deficits). Following this evaluation, the participant’s case was reviewed in a weekly meeting attended by study clinicians and investigators to reach consensus regarding eligibility. Based on our experience in prior studies of back-related leg pain [3], this consensus meeting is highly valuable for ensuring participants leg symptoms are back-related and not primarily due to other pathologies (e.g., osteoarthritis of the hip or knee) in addition to making recommendations for potential further diagnostic work up to rule out contraindications to study treatments prior to enrollment. Eligible participants attended a final baseline evaluation to confirm consent, complete patient-reported outcomes, and be randomly assigned to one of the study interventions. All study clinicians and staff involved in screening were blinded to upcoming treatment assignments.

### Randomization

The random allocation sequence was prepared by the study biostatistician using computer generated block randomization (with varying block sizes) stratified by QTF categories for back-related leg pain (QTF categories 2, 3, and 4) with 1:1 randomization. The sequence was programmed into a centralized electronic study database (i.e., REDCap) by a staff member who was not involved in study screening or enrollment procedures. In addition, all investigators were blinded to the allocation schedule (except for the study biostatistician).

### Interventions

Enrolled participants were randomly assigned to up to 12 weeks of 1) Supported Biopsychosocial Self-Management (SBSM) or 2) Medical care. Participants were asked to refrain from non-study provider-based care for their back-related leg pain during the 12-week treatment period. This included the use of prescription medications from non-study providers for pain. They could continue with self-management practices including over-the-counter medications. Interventions were provided on site at an outpatient research clinic or via videoconferencing using Zoom by providers with at least three years of clinical experience.

All participants received a booklet, entitled ‘Back in Action’ with standardized information about causes and prognosis of BRLP, as well as basic self-management practices (e.g., use of over-the-counter medications, keeping active, heat and cold). Following the 12-week treatment period, participants who experienced an aggravation of symptoms [50] had the option of returning for additional visits in their assigned intervention until their participation in the study ended (6 months after enrollment). Study visits were video recorded with the participant’s consent and 10% were assessed for fidelity by study investigators. See Appendix Table 1 for a description of the study interventions using the Template for Intervention Description and Replication checklist [51].

**Table 1:** Feasibility measures and performance guided by the RE-AIM framework recruitment

Recruitment	Goals: ≥ 40 screened/month (50% female, 25% minoritized racial or ethnic populations)
Performance: 617 total screens in 8 months; > 100 screened/month during the 3 months of peak study screening activity; 69% female, 27% from minoritized racial or ethnic populations	
Enrollment	Goals: ≥ 8 enrolled/month (50% female, 25% minoritized racial or ethnic populations)
Performance: 35 enrolled in 4-month period (enrolled/month ranged from 7 to 11 during that period; 60% female, 19% from minoritized racial or ethnic populations)	
Intervention acceptability, credibility, safety	Goals: ≤ 10% never receive any treatment; ≤ 10% receive prohibited treatments during 12-week intervention phase (contamination); ≥ 80% satisfied with SBSM treatment; no pre-specified safety goals
Performance: 41 of 42 enrolled participants received treatment (98%); 2 of the 42 enrolled participants (5%) sought prohibited treatments outside the study during the 12-week intervention phase (1 SBSM participant had a massage visit, and 1 MC participant visited a chiropractor); 85% satisfied with SBSM treatment	
Participant adherence	Goals: ≥ 80% participants attend required sessions (SBSM = 6; MC = 2); ≥ 70% of SBSM participants report participation in home practices; ≥ 70% of MC participants report taking medications as prescribed
Performance: 93% of participants attended required sessions (39/42); 95% of SBSM participants reported engaging in home practices at 3 months (19/20); 77% of MC participants reported taking medications as prescribed (17/22)	
Provider fidelity	Targets: Providers deliver 100% of required intervention activities on ≥ 70% of visits
Performance: SBSM providers delivered all required intervention activities on 72% of visits (111/155). All but one of the required activities (encouragement to use daily logs) were performed at 93% of sessions (n = 144/155); MC providers delivered 100% of required intervention activities on 99% of visits (79/80)	
Data collection	Targets: ≥ 85% of participants complete 3 month follow up; ≥ 80% of participants complete 6 month follow up; ≥ 80% of weekly pain severity and frequency surveys completed
Performance: 90% of participants completed 3 and 6 month follow up; 86% of weekly pain severity and frequency surveys were completed with 81% of participants completing ≥ 80% or at least 21 of the 26 weekly surveys	

## Supported biopsychosocial self-management (SBSM)

**Fig. 1:**
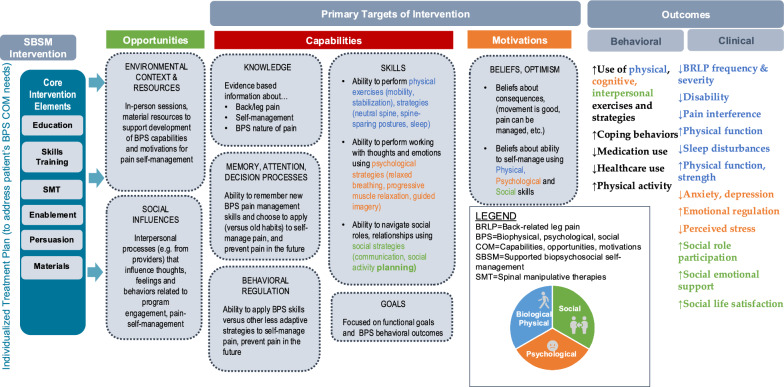
Underlying conceptual framework supported biopsychosocial self- management

The multi-modal SBSM approach was designed using intervention mapping, a systematic process for developing complex interventions that aligns theory and the best available evidence with stakeholder needs and desired outcomes [52]. The well-established Behavior Change Wheel [53] and BPS [11, 54] theoretical models were applied given the goals of addressing BRLP from a more comprehensive perspective, as well as addressing self-management as a targeted behavior. An advantage of the Behavior Change Wheel model is that is represents a synthesis of 19 behavioral theoretical frameworks and is more comprehensive than a single theory driven model. This model posits that to achieve a desired behavior interventions must address individuals’ capability, opportunity, and motivational needs. The goal of SBSM was to provide patients the opportunities and resources to develop their capabilities and motivations to engage in healthy pain self-management behaviors (e.g., increased movement, decreased medication use, etc.). Figure 1 provides an overview of the SBSM intervention’s underlying conceptual framework which illustrates the primary targeted needs (from a behavioral perspective) and outcomes. SBSM was provided by licensed PTs and DCs.

**Fig. 2:**
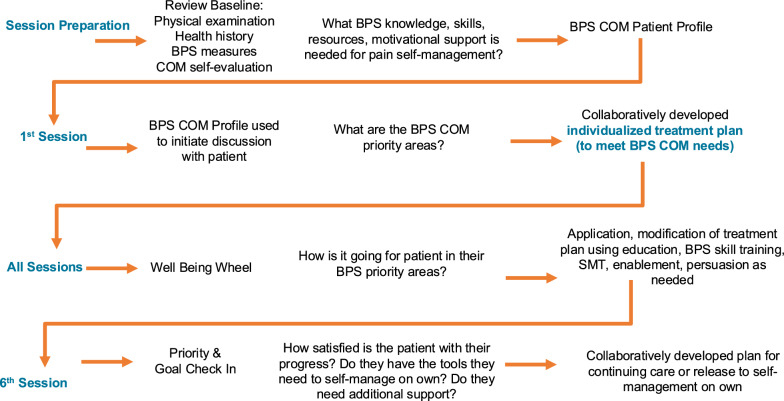
SBSM overview

SBSM consisted of 6–12, 60-min, one-on-one sessions with a provider. Providers were trained to deliver session activities as outlined in Fig. 2; additional details are also provided in Appendix Table 4 using the Template for Intervention Description and Replication Checklist.

- *Session preparation* Prior to the first session, the provider performed a needs assessment by reviewing the patient’s BPS intake measures and physical examination findings, in addition to the patient’s Capability, Opportunity, and Motivation (COM) self-evaluation [53] illustrating what knowledge, skills, resources and motivational support they felt they needed to engage in self-management. The provider used a summary template programed in REDCap to compile the information into a standardized BPS COM patient profile.
- *Session 1* At the first session, an overview of the program was provided. The provider reviewed findings from the participant’s baseline history and physical exam, followed by information regarding common causes of pain, the intersections of biophysical, psychological, and social factors, and the importance of the mind–body connection. In addition, the provider used the participant’s BPS COM profile to initiate a collaborative discussion regarding an individualized treatment plan which included priority areas, short-term goals, and potential intervention strategies for achieving them.
- *All Sessions* Session treatment activities focused on discussing patients’ views of how they were doing from a BPS perspective using a Wellbeing Wheel for orientation, a check in on priorities and goals, and identification of strategies for meeting goals, including prioritization of activities for that day’s session. The sessions provided the opportunity to deliver the core intervention elements (as defined by the Behavior Change Wheel Model) of education, skill training, SMT, enablement, persuasion as needed, to address patients’ capability and motivational self-management needs [53].
- *Education*, using evidence-based information about chronic pain, BRLP, biopsychosocial risk factors, and self-management to enhance patients’ knowledge [18].
- *BPS self-management skill training* in the following strategies and exercises based on individual need: physical exercises (e.g. postural, strength, stabilization and mobility exercises); [3, 55] psychological strategies (e.g. progressive muscle relaxation, relaxed breathing, guided imagery, pacing, relaxation, problem solving, and cognitive restructuring) [56–58]; and social strategies, including pleasant activity planning with a social focus, and communication techniques for navigating relationships (e.g. work, family, friends) to garner support for self-sufficiency. Specific behavior change techniques (BCTs) used as part of skill training included instructions, demonstrations, practice and rehearsal, feedback, self-monitoring and graded progressions [53, 59].
- *Spinal manipulative therapy (SMT)* was applied as a “bridge therapy” as indicated, to support patients’ abilities to engage in the skill development described above. An important facilitator to engagement in self-management for pain is the belief that the condition can improve [60, 61]. SMT can produce immediate symptom changes [62, 63] and may facilitate engagement in self-management behaviors as it shows that improvement is possible. SMT included soft-tissue work (e.g. cross-fiber stretch, light friction massage, etc.), mobilization (low velocity, low–high amplitude passive movements) and manipulation (high velocity, low amplitude thrust) [3]. SMT was applied to the lower thoracic/lumbar spine or sacroiliac joints as indicated by physical exam. The type and frequency of SMT was individualized based on participant need. All participants received SMT during at least one session as it was a core intervention element.
- *Enablement* was applied to encourage engagement in self-management, and if needed, to address patients’ unhelpful beliefs about their capabilities to self-manage BRLP and overall health. Examples of specific BCTs used as part of enablement included emotional support provided by the provider, value-based functional goal setting and goal review, action planning/problem solving, and monitoring [53, 59].
- *Persuasion* was used as needed to influence patients BRLP beliefs, optimism, and motivation which are important for the adaptation of healthy pain coping behaviors. The following BCTs were integrated into the intervention: verbal persuasion, focus on past successes, and framing/reframing [53, 59].
- *Materials* were provided to patients to support their self-management, including a workbook with educational materials, worksheets, and progress tracking sheets and a website with video and audio recordings of exercises and strategies addressed in the session skill training (see below).
- *Session 6* At the sixth session the provider used a Priority & Goal Check-In Sheet to initiate a discussion with the patient regarding their satisfaction with their progress, barriers and facilitators to progress and goals. This included an assessment of their confidence level in continuing to use BPS skills and continuing to work towards their valued activities and goals on their own. Needs and preferences for additional clinician support (e.g. more sessions) were also explored, resulting in a decision to continue with care, reduce frequency of care, or release to self-management on own.

### SBSM provider training and support

We developed a training and support system to address providers’ own capability, opportunity and motivational needs and overcome barriers, to effectively delivering SBSM, based on provider input from previous trials [64–66], assessment of providers prior to the feasibility study, and the emerging literature on clinicians’ behavioral related needs for supporting patient self-management [45, 53]. Provider training and support was designed to facilitate intervention fidelity, and ensure more reproducible processes supporting patient self-management.

*Provider capabilities* (knowledge, skills) were addressed through 40 h of education and training that included instructions, demonstrations, and practice (with feedback) on implementing the core intervention elements, as well as the use of a core set of behavior change techniques (BCTs) and motivational communication techniques (CTs) [53, 59] to effectively support the patient in developing adaptive pain self-management behaviors and foster an effective therapeutic alliance [67)] This was supplemented by ongoing training, enablement, support and persuasion from study investigators responsible for the intervention (Evans, Greco, Leininger) through bi-weekly group meetings where barriers and facilitators to delivering the intervention for specific cases were discussed.

*Provider opportunities and resources* were addressed by providing tools and materials to navigate the session activities. These included an electronic (REDCap) BPS COM profile summary template to help assess patients’ needs and match them to the appropriate intervention elements. In addition, providers used structured treatment notes which pulled forward previous visit information and guided clinicians through required activities and documentation. Providers were also given an easy to follow ‘Clinician Guide’ with checklists for each visit, worksheets (e.g. Well Being Wheel, Priority & Goal Check In) to guide collaborative discussions and decision making, and reminders and suggestions for using BCTs and CTs when presented with different circumstances.

*Provider motivations* were addressed by prompting clinicians to identify barriers and facilitators to delivering the intervention at each session. They were also asked to reflect on their own beliefs about the consequences of delivering intervention elements (e.g. physical exercises, psychological or social strategies) and confidence in their ability to deliver the interventions.

## Medical care

Medical care was primarily medication management as this is the standard first-line approach for back-related leg pain in primary care. Choice of medications was informed by the evidence [15, 68, 69].

- Nonsteroidal anti-inflammatory drugs (NSAIDs) were used as a first-line approach.
- Second-line medications included systemic corticosteroids, skeletal muscle relaxants, acetaminophen, benzodiazepines, antiseizure medications, lidocaine patches, serotonin norepinephrine reuptake inhibitors, tricyclic antidepressants and weak opioids (e.g. Tramadol, Tylenol with Codeine) for participants unable to tolerate or unresponsive to first-line medications.
- Strong opioids were not allowed, as the CDC recommendations prefer non-opioid medications for chronic pain and there is a lack of evidence regarding their use for BRLP [68, 70].

Medical care included 2 or more visits with a study Nurse Practitioner. Decisions regarding medication selection were made collaboratively between the provider and patient after a discussion of the potential risks, benefits, past experience, and preferences for different medications. Required intervention activities at each visit included the assessment for medication need and following the protocol for first and second-line medications.

### Data collection

Data collection for the feasibility study included study flow data (e.g., number of participants screened, data collection rates), participant surveys, provider documentation of intervention activities, provider views of the SBSM intervention, and video recordings of intervention sessions. Data was primarily collected using electronic data capture through REDCap, a secure web application for building and managing online surveys and databases [71].

### Feasibility outcomes

Feasibility outcomes and a priori targets were defined for recruitment, enrollment, intervention acceptability and credibility, participant adherence, provider fidelity, and data collection (Table 1).

Choice of feasibility measures were guided by the RE-AIM framework, to identify factors that could impact the future full scale trial’s success, as well as implementation of the experimental intervention in clinical practice, should it prove effective. Consistent with the study’s objective, emphasis was placed on the “reach” and “implementation” RE-AIM domains, using mixed methods data collection to gather important contextual data from study participants and providers [49]. Measures for “reach” are detailed under recruitment and enrollment feasibility and measures for “implementation” are detailed under participant adherence and provider fidelity. These measures were collected for feasibility assessment and protocol optimization for the full-scale trial, rather than assessing the broader implementation context that is often associated with the framework.

*Recruitment and enrollment feasibility* were assessed using screening and enrollment rates, reasons for exclusion or choosing not to participate, recruitment sources, and demographic and clinical characteristics of screened and enrolled participants (See description of demographic and clinical measures under data collection feasibility). A high priority for this feasibility study was to answer key recruitment and enrollment feasibility questions related to our reach [49]. This included whether we could enroll sufficient numbers of people with BRLP, including those often underrepresented in research because of intersecting social factors, including race, ethnicity, education, and income [72]. Further, given the tendency for individuals to have strong preferences for one intervention over another [73], it was critical to establish whether interested individuals were willing to accept being randomized to medical care which could appear less appealing than a newer, supported self-management approach.

*Intervention acceptability, credibility, and safety* was assessed using the proportion of enrolled participants never receiving treatment, satisfied with treatment, receiving prohibited treatments during the intervention phase, and experiencing adverse events. Potential adverse events were systematically assessed at each intervention visit and during monthly participant surveys.

*Participant adherence* measures included attending the required # of sessions, participating in home self-management practices (SBSM group), and taking medications as directed (Medical care group). For SBSM participants, we asked about their level of satisfaction for each of the intervention components and resources (e.g., SMT, physical exercises, mind–body strategies, workbook). We also asked about overall views of the program including barriers and facilitators to BRLP self-management and how well the intervention met their individual capability, opportunity, and motivational needs using a combination of closed and open-ended survey questions. This included assessing participants’ receptivity to PTs and DCs providing a whole person approach to care, since standard practice currently focuses on biophysical treatments (e.g., exercise, spinal manipulation).

*Provider fidelity* was assessed using data from study intervention visits detailing what activities were performed in addition to reviewing session video recordings. We collected information from SBSM providers at every visit including satisfaction with their overall ability to conduct the session, if they had sufficient knowledge, skills, and resources to conduct the session confidently and competently, and if they believed the activities were appropriate using a mix of closed and open-ended survey questions. Provider views on the SBSM intervention were also collected during a post-study focus group interview. Participant and provider views of the SBSM intervention will be reported in a separate manuscript along with findings from reviewing session video recordings for provider fidelity.

*Data collection* feasibility was assessed using completion rates for surveys collecting clinical, behavioral, and potential mediating outcomes for the full-scale trial detailed below. This included completion rates for weekly and monthly surveys over the six-month study duration in addition to completion rates at months 3 and 6 which included all clinical, behavioral, and potential mediating outcomes. Weekly surveys included BRLP and LBP frequency and intensity and monthly surveys included disability, PROMIS-29 + 2, medication and healthcare use, productivity loss, and adverse event measures. Details regarding the data collection schedule for clinical, behavioral, and potential mediating outcomes are provided in Appendix Table 5.

### Sample size

The study was designed to assess feasibility for a full-scale trial and was not powered to detect important differences in clinical outcomes. It was informed by previous pilot studies performed by the investigative team where approximately 15–20 participants per group was sufficient for identifying potential important issues with recruitment and enrollment procedures, intervention protocols, outcome measures, and data collection rates to inform the feasibility of a larger clinical trial.

### Analyses

Quantitative feasibility outcomes were analyzed using descriptive statistics including means, medians, and frequencies. No within or between group statistical analyses of clinical outcomes were planned or performed due to the focus on feasibility. A rapid deductive, directed content analysis was conducted for qualitative data from open ended survey questions and the post-study focus group interview with providers. Rapid approaches can balance rigor with efficiency, yielding timely and meaningful evaluation of stakeholder perspectives [74, 75].

## Feasibility results

### Recruitment and enrollment feasibility

Enrollment of participants was originally anticipated to last 7 months, with two months of increasing enrollment before reaching our target of enrolling 8 participants per month. We enrolled a total of 42 participants over a 6-month period (Late January to Early August 2022). After the initial two-months of increasing enrollment, we were able to recruit between 7 and 11 participants per month for the remainder of the study. We screened 617 total participants for the study. The number of participants screened per month ranged from 82 to 278 after the initial two months of increasing enrollment which was more than double our original target screening rate for feasibility.

**Fig. 3:**
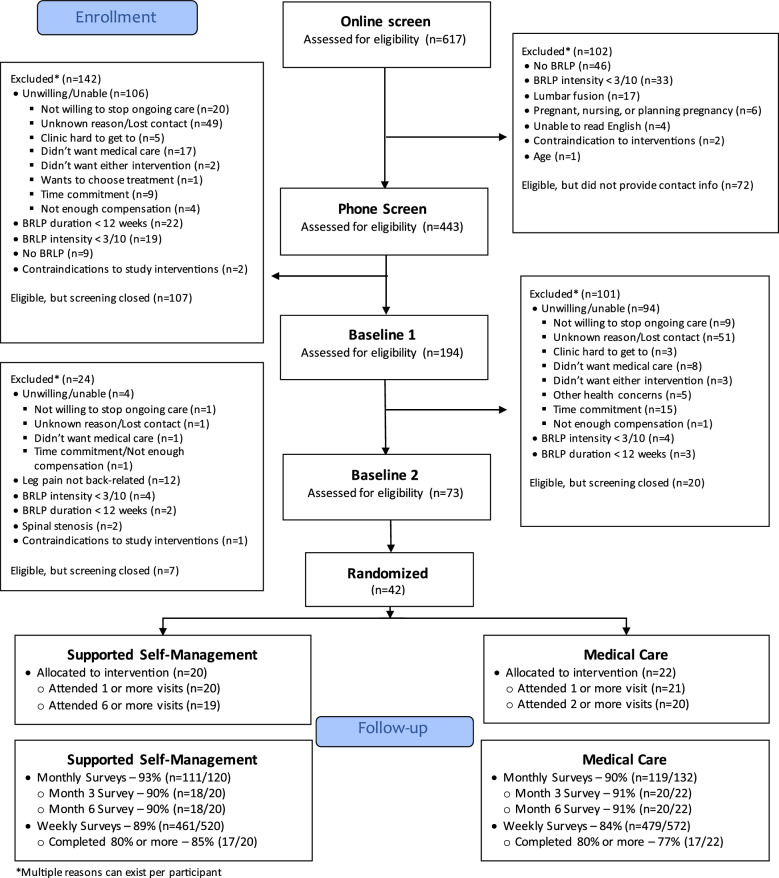
Consort diagram

Of the 617 total participants, 134 were undergoing screening when we met our sample size goal and closed study enrollment. Overall, we enrolled 42 of the 441 participants who completed screening (approximately 10%). Reasons for exclusion at study screening visits are displayed in Fig. 3. The most common reasons for exclusion were participants not responding to contact attempts after showing initial interest (n = 101, 23%), failure to provide contact info (n = 72, 16%), back pain without leg pain or leg pain that wasn’t back-related (n = 67, 15%), and BRLP intensity that was less than 3/10 in the past week (n = 60, 14%). Less common reasons for exclusion included not wanting to stop ongoing BRLP care (n = 30, 7%), lack of chronic BRLP (n = 27, 6%), not wanting to receive medical care (n = 26, 6%), and the time commitment (n = 25, 6%).

The most commonly reported recruitment sources were Facebook advertisements (n = 319, 52%), referrals from other studies by the investigative team (n = 166, 27%), referrals from friends or family members (n = 56, 9%), and postcard mailings (n = 30, 5%). Demographic and clinical characteristics were similar between screened and enrolled participants (Table 2 and Appendix Table 6). Overall, approximately two-thirds of enrolled participants were from NIH-designated U.S. health disparity populations that includes racial or ethnic minoritized populations, sexual and gender minorities, or socioeconomically disadvantaged populations. Participants ranged in age from 22 to 79 years (mean age of 52.9 years) with 21% being 65 or older. Approximately 70% of screened and 60% of enrolled participants reported female sex at birth. Over three-quarters of participants were White, non-Hispanic (79%), while 10% were Black or African American and 7% were Asian. In terms of socioeconomic status, nearly a quarter of participants reported an annual household income less than $45,000 per year and 40% did not have a 4-year college degree. Approximately 10% of participants reported experiencing food insecurity, 30% had delayed medical care in the past year due to cost, and 4% did not have health insurance.

**Table 2:** Demographic and clinical characteristics for screened and enrolled participants

	Screened participants: (N = 617 unless noted otherwise)	Enrolled: Participants: (N = 42)
From population experiencing health disparities*, n (%)	385 (62%)	28 (67%)
*Age, Mean (SD)*	53.4 (15.8)	52.9 (14.2)
*Age categories, n (%)*		
0 to 17	1 (0.2%)	0 (0%)
18 to 34	95 (15%)	5 (12%)
35 to 49	146 (24%)	14 (33%)
50 to 64	191 (31%)	14 (33%)
65 to 79	171 (28%)	9 (21%)
*Sex at birth, n (%)*		
Female	414 (69%)	25 (59.5%)
Male	181 (30%)	17 (40.5%)
*Education, n (%)*	N = 597	
No high school diploma	12 (2%)	1 (2%)
High school graduate or GED	40 (6%)	4 (10%)
Some college, no degree	117 (19%)	6 (14%)
Associate degree	78 (13%)	6 (14%)
Bachelor’s degree	173 (28%)	15 (36%)
Master’s degree	127 (21%)	10 (24%)
*Smoking History, n (%)*	N = 90	
Never	51 (57%)	25 (59.5%)
Current	8 (9%)	4 (9.5%)
Former	31 (34%)	13 (31%)
*Clinical Characteristics*	N = 90	
BRLP duration in weeks, median (IQR)	104 (0 to 1,560)	156 (52 to 312)
*STarTBack, n (%)*	N = 90	
Low risk	27 (30%)	11 (26%)
Medium risk	48 (53%)	23 (55%)
High risk	15 (17%)	8 (19%)
*Pain Detect Score*	N = 90	
< 13 – Unlikely neuropathic	46 (51%)	17 (40%)
13–18 – Unclear if neuropathic	30 (33%)	15 (36%)
> 18 – Likely neuropathic	14 (16%)	10 (24%)
*Perceived risk BRLP will remain persistent (0 to 10 with higher scores indicating larger risk), mean (SD)*	7.5 (2.2)	7.5 (2.1)
*Quebec Task Force Classification, n (%)*	N = 76	
2—Pain above the knee	26 (34%)	13 (31%)
3—Pain below the knee	29 (38%)	15 (36%)
4—Pain with neurological signs	14 (18%)	14 (33%)
No BRLP or unclear classification	7 (9%)	0 (0%)

^*^Population Experiencing Health Disparities defined by at least one of the following: biological sex other than male or female; gender identity other than man or woman; sexual orientation other than straight; Hispanic, Latino, or Spanish ethnicity; Non-White race; education level lower than Bachelor's degree; annual household income < $45,000; food insecurity; lack of health insurance

For clinical characteristics, the median episode of BRLP had lasted 3 years and a third of participants had accompanying neurological signs including dermatomal muscle weakness, sensory deficits, or diminished reflexes. Nearly three-quarters of participants were classified as medium or high risk on the STarTBack screening tool. Appendix Table 7 details the baseline distribution of clinical, behavioral, and potential mediating outcomes by treatment group. Participant’s BRLP was present 5 days per week with moderate intensity (5 on 0–10 scale) and disability (13 on 0–23 scale). Over two-thirds of participants were using medications to manage their BRLP, most commonly NSAIDs and non-narcotic analgesics (e.g. acetaminophen). Roughly two-thirds reported reduced work productivity in the past month for both paid and unpaid work. Satisfaction with specific life domains was typically high, but was rated lower for their health, work, leisure activities, and energy level. Common coping strategies were guarding, resting, task persistence, exercise, and using coping self-statements. Treatment expectations were high for both interventions and similar between groups.

### Intervention acceptability, credibility, and safety

Overall, intervention acceptability and credibility were excellent. A total of 41 of the 42 enrolled participants (98%) accepted their randomized treatment and attended at least one treatment session. Further, only two of the 42 enrolled participants (5%) received prohibited treatments outside the study during the 12-week intervention phase. One participant in the SBSM group visited a massage therapist for their BRLP, and one participant in the medical care group visited a chiropractor. In terms of satisfaction with the study interventions, 85% of participants in the SBSM group reported being satisfied with the intervention overall.

Expected adverse events during the intervention period were mild to moderate and self-limiting. Two participants in the medical care group reported serious adverse events due to inpatient hospitalization for fracture injuries from a fall. Both events were unexpected, occurred after the intervention period, and were not related to study interventions. One participant fractured their leg while the other sustained fractures of their cervical and thoracic spine.

### Participant adherence

For SBSM, 19 of the 20 participants attended 6 or more sessions and did not withdraw from treatment (95%). One participant attended 3 sessions before withdrawing from treatment due to a lack of improvement. The total number of SBSM sessions attended ranged from 3 to 10 with 75% of participants attending between 7 and 9 sessions. At the end of the 12-week intervention period, 95% of SBSM participants reported engaging in home practice of SBSM physical or mind–body exercises in the past week (see Table 3). For medical care, 20 of the 22 participants attended 2 or more visits and did not withdraw from treatment (91%). One participant attended one visit, and another did not attend any visits before withdrawing from treatment for unknown reasons. The total number of medical care visits attended ranged from 0 to 7 with over 75% of participants attending between 2 and 5 visits. Throughout the intervention period, 17 of the 22 medical care participants (77%) reported taking medications as prescribed.

**Table 3:** Self-reported use of SBSM exercises/strategies at the end of the intervention period (Number of days used in the past week)

	0 days or unknown	1–3 days	4–5 days	6–7 days
Physical exercises	1 (5%)	1 (5%)	6 (30%)	12 (60%)
Mind–body strategies	1 (5%)	6 (30%)	6 (30%)	7 (35%)
Posture exercises	2 (10%)	1 (5%)	6 (30%)	11 (55%)
Sleep strategies	9 (45%)	1 (5%)	3 (15%)	7 (35%)
Communication strategies	9 (45%)	7 (35%)	4 (20%)	0 (0%)

### Provider fidelity

Provider fidelity to the SBSM intervention was good. All but one of the required activities were performed at 93% of sessions (n = 144/155). Encouragement to use the daily log for tracking self-management strategy use and potential barriers was not performed in 27% of sessions (n = 42/155). Medical care providers delivered the required intervention activities on 99% of visits (n = 79/80). Post study debriefing provided insight into the providers’ experience, including challenges to intervention delivery which will be important to address in a future full-scale trial. This included difficulty assimilating and prioritizing the large amount of BPS data required to develop an individualized treatment plan. They also identified a need for additional resources and tools for supporting clinical decisions regarding which BPS needs should be prioritized over the course of treatment. Providers also mentioned some difficulty navigating the shared decision-making process and shifting from a traditional provider driven approach. Finally, supporting participants release from care to self-management was difficult in some cases, and further tools and training were requested to support this. A separate manuscript will report on more detailed findings from mixed methods analyses of participant and provider data regarding the SBSM intervention.

### Data collection feasibility

Overall, completion rates for monthly surveys were high with participants completing 91% of all monthly surveys (n = 230/252) and 90% of surveys at months 3 and 6 (n = 38/42). Completion rates for monthly surveys were similar between groups. For weekly surveys, participants completed a total of 86% of all surveys (n = 940/1092), with 81% of participants completing at least 80% of their weekly surveys. The percentage of participants completing at least 80% of their weekly surveys was 85% in the SBSM group (n = 17/20) compared to 77% in the Medical Care group (n = 17/22). Among the completed surveys, no outcome measures had any missing data. Completion rates by intervention group are detailed in Fig. 3.

## Discussion

This feasibility study provided an important opportunity to develop and assess key study areas that could affect a future full scale trial’s methodological quality, and eventual implementation of the experimental intervention. Overall, we demonstrated that a full-scale trial comparing a whole person self-management intervention to medical care is feasible to conduct. We also learned about important issues and areas for optimization that could affect long term success in both research and clinical practice.

### Recruitment and enrollment

The pilot study met or exceeded nearly all our pre-specified goals for recruitment and enrollment. This was a promising finding given we anticipated recruitment, and enrollment would be particularly challenging as the study opened during a large wave of COVID-19 infections in December 2021. The number of participants screened per month was more than double what we anticipated would be necessary to reach our enrollment goals. Only 6% of individuals decided they did not want to participate due to a strong preference for the SBSM intervention over medical care, and only 1 participant did not initiate medical treatment. Importantly, the study had a better representation of individuals from minoritized racial or ethnic populations compared to many studies in the back pain field [72, 76, 77]. We also enrolled a large percentage of individuals from populations that experience health disparities due to other factors such as education, income, gender identity, sexual orientation, healthcare access, or food insecurity. We attribute this to spending more time in diverse communities in the Minneapolis/St. Paul metro region and working together with a Community Advisory Team to guide our engagement efforts. Further, the use of the PhenX Toolkit’s social determinants of health measures was pivotal for accurately and more comprehensively describing the diversity of the population we were reaching.

While we were largely successful with recruitment and enrollment, the pilot study afforded us the opportunity to also identify issues that will need to be addressed in the future trial. For example, we did fall short of our planned enrollment goals for race and ethnicity (19% actual versus 25% planned). The short time frame for recruiting and enrolling participants into the pilot study proved to be a challenge, especially for initiating, monitoring, and adapting recruitment and engagement methods within diverse communities where building trust is a necessary first step requiring substantial commitment and time [78–81]. To address this issue, the study team has made concerted efforts to engage in the community, even in the gap period between the pilot study and full-scale trial. The full-scale trial anticipates using similar recruitment strategies with a focus on community rather than healthcare clinic based strategies.

### Intervention engagement and fidelity

Overall, participant engagement with study interventions was high with over 90% attending the minimum number of visits (95% for SBSM, 91% for Medical care). We credit the application of several engagement strategies including clear outline and discussion of expectations for the interventions during informed consent, flexibility in the intervention delivery format (in-person or videoconference) and timing for attending visits (availability of early morning and evening appointments), and staff support for providing reminders, monitoring attendance, and troubleshooting individual barriers to participation. Engagement in SBSM home practices was also very high (95%) which is particularly promising given past studies finding half to two-thirds of participants typically don’t engage in self-management home practices [22, 82]. Importantly, engagement in physical exercise and psychologically oriented strategies (e.g. guided imagery, relaxed breathing) were similar in the SBSM group providing support for participants’ receptivity to a whole person approach to care from traditionally biophysically orientated providers (PT’s and DC’s). With regards to medical care, over 75% of participants reported taking medications as prescribed and two-thirds were satisfied with their care, which is similar to findings from another study assessing medical care for chronic BRLP [83].

Overall, fidelity for completing required intervention activities was very good with providers completing all but one of the required activities (encouraging use of a daily log) on over 90% of visits. Providers noted some challenges implementing the SBSM intervention. This included the need to process large amounts of BPS data, prioritize different self-management skills, and engage in shared decision making. Additional training, tools, and resources have been identified to ensure providers are further supported during the full-scale trial.

### Follow up

Overall, data collection rates were high for both weekly (86%) and monthly surveys (91%) and exceeded our pre-specified goals. We successfully implemented several strategies to support data collection processes which included a clear discussion of the importance of data collection to trial validity during the consent process, flexible format for completion (self-completion using web platform via email or text message invitation, phone call with blinded staff), and routine monitoring and reminders for incomplete surveys.

### Strengths and limitations

Smaller pilot studies are invaluable for establishing feasibility. Too often however, they are inappropriately focused on reporting differences in clinical outcomes and hypothesis testing, with attempts to draw conclusions, which the study was not designed to address [46]. A strength of this study is that it avoided these pitfalls and was conducted and reported in a manner that maximizes utility within the confines of its design to ensure methods and protocols were adequately tested before a larger, more resource intensive study is undertaken. Importantly, we used pre-defined feasibility outcomes that will support the methodological rigor of the future effectiveness trial and identified barriers and challenges from the participant and provider viewpoints, which illustrated areas for protocol refinement. While differences in clinical outcomes are intentionally not reported, we did find that both groups experienced meaningful improvements in clinical outcomes such as back related leg pain intensity, frequency, and disability. Specific outcomes are not reported to avoid inappropriate inclusion in systematic reviews. Another important strength of this study is the systematic application of a comprehensive behavioral model to design a clearly articulated intervention (Appendix Table 4) specifying active intervention elements and how providers can deliver them using patient-centered behavior change techniques [52, 53]. This can facilitate future translation and increase the ability to train providers to better support pain self-management which remains a challenge in musculoskeletal pain practice [84–87].

Limitations included the lack of control for time and attention between SBSM and medical care and lack of blinding for participants and intervention providers. We chose medical care as the comparison group over a time and attention control to maximize the potential impact of the future full-scale trial. The majority of non-surgical BRLP cases (approximately 70%) are managed by primary care physicians [88] and evidence-based guideline recommendations for BRLP are lacking due to the limited amount of high-quality research comparing non-surgical treatments [14, 15, 89].

## Conclusions

Overall, we demonstrated that it’s feasible to conduct a full-scale randomized trial comparing a whole-person supported self-management intervention to medical care for chronic back-related leg pain. We met pre-specified targets for recruitment, enrollment, intervention acceptability and credibility, participant attendance and home practice, intervention fidelity, and data collection. The feasibility study also identified important areas for optimization . The planned full-scale SUPPORT trial will address important evidence gaps by comparing the whole-person supported self-management approach to pharmacological medical care, which is the most common approach for chronic BRLP in the U.S.

## Data Availability

Study data are available from the corresponding author by reasonable request.

## Appendix

See Tables 4, 5, 6 and 7

**Table 4:** Description of interventions based on the template for intervention description and replication

	Supported biopsychosocial self-management (SBSM)	Medical care
Rationale and goal	Rationale: Back-related leg pain (BRLP) is a complex condition impacted by interrelated physical, psychological, and social risk factors	Rationale: Medications are recommended by evidence-based guidelines for LBP and BRLP; they are used by common front-line providers like physicians and advanced practice providers; it is well suited to serve as a comparison intervention
	Goal: to provide patients the opportunities and resources to develop the capabilities and motivation to engage in healthy pain self-management behaviors	Goal: to reduce BRLP symptoms and provide care as it would typically be delivered in primary care settings
Participant materials	Back In Action booklet with educational material regarding the causes, prognosis, and general self-management tips for BRLP	Back In Action booklet with educational material regarding the causes, prognosis, and general self-management tips for BRLP
	Workbook	
	Website with video and audio recordings	
Clinician materials	Manual of operations	Manual of operations
	BPS COM profile for summarizing individual’s BPS strengths and needs elated to guide individualized care	
	Clinician guide with tools to guide discussions (e.g. Priority & Goal Check In, Wellbeing Wheel worksheets), session checklists and prompts and cues to facilitate delivery of required activities in a supportive manner	Treatment visit forms with session checklists to facilitate delivery of required activities and protocol compliance
	Treatment visit forms with session checklists to facilitate delivery of required activities and protocol compliance	
Procedures	Needs assessment, individualized treatment plan (also see Tailoring and Individualization below)	–Needs assessment, individualized treatment plan
	BPS Self-Management Skills Training including physical exercises; psychological and social strategies	
	Spinal manipulation therapy (SMT) as indicated to manage symptoms	
	Enablement as indicated to improve patients’ unhelpful beliefs	Medications (1st line: Non-steroidal anti-inflammatory drugs; 2nd line: systemic corticosteroids, skeletal muscle relaxants, acetaminophen, benzodiazepines, antiseizure medications, lidocaine patches serotonin and norepinephrine reuptake inhibitors, tricyclic antidepressants, and weak opioids (e.g. Tramadol, Tylenol with Codeine) for participants unable to tolerate or unresponsive to first-line medications
	Education using evidence-based information about chronic pain, BRLP, biopsychosocial risk factors, and self-management	
	Persuasion as needed to influence patients BRLP beliefs, optimism, and motivation	
Intervention providers and training	Licensed physical therapists, chiropractors	Licensed physicians, nurse practitioners
	3 years of clinical experience	3 years of clinical experience
	40 h of initial training; bi-weekly 1 h group clinician meetings to facilitate address their own capability, opportunity and motivational needs and ensure intervention fidelity; additional refresher training as needed	2 h initial training; additional refresher training as needed
Mode of delivery	One-to-one	One-to-one
	In person or videoconference	In person or videoconference
		Telephone visits allowed after 1st visit
Locations	University-affiliated research clinic or by videoconference	University-affiliated research clinic or by videoconference
Frequency, time period, schedule, and duration, intensity, or dose of intervention	6 to 12 visits over 12 weeks	2 or more visits over 12 weeks
	Visits typically occurred weekly	Visits did not have a set schedule
	Each visit lasted between 45–60 min	Each visit 15–30 min long
	Choice and dosing of mind–body and physical exercises was individualized	Choice and dosing of medications was individualized
Tailoring and individualization	Number and frequency of visits depends on needs after minimum of 6 visits reached; release to self-management determined by Priority & Goal Check In worksheet assessing confidence in self-management	Frequency and number of visits depends on needs after minimum of 2 visits reached
	Education: information reiterated, and supplemental information on sleep, communication, physical activity, and tips for pain management or tackling barriers to exercise presented if indicated	
	Training: home exercise plan including practice of physical exercises and psychological ‘mind–body’ strategies tailored to needs, goals and abilities	Medication(s) prescribed based upon participant’s prior history and preferences and clinician judgment
	Enablement/support: customized to patients needs related to training goals and general and emotional support	
	Persuasion: verbal persuasion used as needed to stimulate action	Additional emphasis on information from Back in Action booklet per individual needs
	Additional emphasis on information from Back in Action booklet per individual needs	
Modifications during Study	None	None
Adherence or fidelity assessments	Review of study treatment visit documentation for completion of required activities	Review of study treatment visit documentation for completion of required activities
	Review of video recordings for a random sample of 10% of study visits	Review of video recordings for a random sample of 10% of study visits

**Table 5:** Data collection schedule

	BL	3 m	6 m
Demographics (PhenX Toolkit) [90], STarT Back [91], QTF [92]	X		
*Clinical outcome measures*			
Pain intensity (BRLP and LBP) [93]	X	Weekly	
Pain frequency (BRLP and LBP) [94]	X	Weekly	
Roland morris disability questionnaire [95]	X	Monthly	
Promis-29 + 2 (physical function, pain interference, fatigue, sleep disturbance, anxiety, depression, participation in social roles, cognition) [96–98]	X	Monthly	
Adverse events		Monthly	
Productivity loss [99]	X	Monthly	
Overall Improvement [100] & Satisfaction [101]		X	X
Social emotional support [102]	X	X	X
Domain specific life satisfaction [103]	X	X	X
Perceived stress [104]	X	X	X
*Behavioral outcome measures*			
Medication use	X	Monthly	
Healthcare utilization	X	Monthly	
Coping behaviors [105]	X	X	X
Use of key SBSM skills		X	X
Physical activity [106]	X	X	X
*Mediating outcomes*			
Beliefs (self-efficacy [107], fear avoidance [108], catastrophizing [109], chronic pain acceptance [110], treatment expectations [111])	X	X	X
Satisfaction (patient-provider connection, environment) [111]		X	

*BL* baseline, *BRLP* back-related leg pain, *LBP* low back pain, *m* month, *QTF* Quebec Task Force Classification, *SBSM* Supported Biopsychosocial Self-Management

**Table 6:** Demographic characteristics for screened and enrolled participants

	Screened participants (N = 597 unless noted otherwise)	Enrolled Participants (N = 42)
*Gender identity, n (%)*		
Man	178 (30%)	17 (40%)
Non-binary	21 (4%)	2 (5%)
Transgender	3 (0.5%)	0 (0%)
Woman	396 (66%)	23 (55%)
*Sexual orientation, n (%)*		
Bisexual	37 (6%)	1 (2%)
Gay	14 (2%)	1 (2%)
Lesbian	15 (3%)	1 (2%)
None of these describe me	18 (3%)	0 (0%)
Straight	486 (81%)	37 (88%)
Prefer not to answer	27 (5%)	2 (5%)
*Hispanic ethnicity, n (%)*		
None	554 (90%)	40 (95%)
Mexican, Mexican American, Chicano	12 (2%)	0 (0%)
Puerto Rican	3 (0.5%)	0 (0%)
Cuban	2 (0.3%)	1 (2%)
Other Hispanic, Latino, or Spanish origin	12 (2%)	0 (0%)
*Race, n (%)*		
White	455 (74%)	33 (79%)
Black or African American	59 (10%)	4 (10%)
American Indian or Alaska Native	12 (2%)	0 (0%)
Chinese	8 (1%)	1 (2%)
Korean	2 (0.3%)	0 (0%)
Filipino	1 (0.2%)	0 (0%)
Vietnamese	1 (0.2%)	0 (0%)
Other Asian	6 (1%)	0 (0%)
Other	15 (2%)	2 (5%)
Prefer not to answer	15 (2%)	1 (2%)
More than one race	21 (3%)	1 (2%)
*Household income, n (%)*		
Less than $25,000	71 (12%)	5 (12%)
$25,000 to $44,999	110 (18%)	5 (12%)
$45,000 to $74,999	127 (21%)	10 (24%)
$75,000 to $119,999	126 (20%)	8 (19%)
$120,000 to $224,999	86 (14%)	5 (12%)
$225,000 or more	25 (4%)	3 (7%)
Prefer not to answer	52 (8%)	6 (14%)
*Health insurance, n (%)*	N = 109	
Employer-sponsored	48 (44%)	24 (47%)
Self-purchased	7 (6%)	4 (8%)
Medicare	28 (26%)	9 (18%)
Medicaid	10 (9%)	4 (8%)
Military-sponsored (e.g., TRICARE, VA)	8 (7%)	4 (8%)
Other	5 (5%)	3 (6%)
None	2 (2%)	2 (4%)
*Food Insecurity, n (%)*	N = 90	
Couldn’t afford balanced meals	6 (7%)	5 (12%)
Food didn’t last	5 (6%)	4 (10%)
Delayed medical care in past 12 months, n (%)	24 (27%)	13 (31%)

**Table 7:** Baseline clinical, behavioral, and potential mediating outcomes by treatment group

	Medical Care	Supported biopsychosocial self-management
Days of BRLP in past week, mean (SD)	5.6 (1.7)	5.0 (1.8)
BRLP intensity (0–10), mean (SD)	5.4 (2.0)	5.2 (1.2)
Days of LBP in past week, mean (SD)	5.5 (1.9)	6.1 (1.5)
LBP intensity (0–10), mean (SD)	5.5 (2.3)	5.7 (1.6)
Low back disability (Roland Morris: 0–23), mean (SD)	12.6 (4.3)	13.1 (4.9)
*PROMIS-29†*		
Pain interference	60.6 (7.2)	58.9 (5.0)
Physical function	39.4 (5.0)	40.7 (5.4)
Sleep disturbance	53.7 (7.6)	51.7 (5.2)
Fatigue	55.6 (9.8)	55.2 (7.1)
Anxiety	52.3 (9.2)	52.6 (8.0)
Depression	49.9 (9.2)	51.6 (7.4)
Ability to participate in social roles and activities	48.7 (10.2)	47.7 (7.1)
Any BRLP medication use in past month, n (%)	14 (64%)	15 (75%)
NSAIDs	12 (55%)	11 (55%)
Non-narcotic analgesics	10 (45%)	8 (40%)
Narcotic analgesics	1 (5%)	0 (0%)
Muscle relaxants	1 (5%)	2 (10%)
Corticosteroids	0 (0%)	0 (0%)
Anti-seizure medications	1 (5%)	2 (10%)
Benzodiazepines	0 (0%)	0 (0%)
Lidocaine pain patches	0 (0%)	1 (5%)
Anti-depressants	1 (5%)	2 (10%)
Missed work in past month for BRLP, n (%)	4 (18%)	1 (5%)
Reduced work in past month for BRLP, n (%)	16 (73%)	10 (50%)
Reduced unpaid work in past month for BRLP, n (%)	17 (77%)	10 (50%)
*Self-efficacy for managing chronic conditions†*		
Managing symptoms	43.7 (6.6)	41.9 (5.9)
Managing daily activities	45.9 (6.5)	44.3 (5.4)
Managing emotions	47.6 (7.7)	49.3 (10.6)
Managing social interactions	46.8 (7.0)	48.9 (7.4)
Chronic Pain Acceptance (0 to 120)	69.2 (19.2)	69.7 (11.3)
Activities engagement sub-scale (0 to 66)	40.1 (10.3)	37.8 (8.4)
Pain willingness subscale (0 to 54)	24.9 (8.4)	24.3 (8.3)
*Life satisfaction (1* = *Not at all to 5* = *Very much)*		
Education	4.0 (0.8)	4.0 (1.1)
Work	3.4 (1.4)	3.6 (1.5)
Spiritual, religious, or philosophical well-being	4.0 (1.0)	4.2 (0.8)
Housing	4.5 (0.7)	4.6 (0.6)
Family life	4.1 (1.1)	4.0 (1.1)
Health	3.0 (1.0)	3.1 (0.8)
Friends and social life	4.0 (0.8)	3.5 (1.1)
Neighborhood	4.0 (1.0)	3.8 (1.2)
Ability to help others	4.1 (0.8)	3.8 (1.1)
Achievement of my goals	3.6 (1.0)	3.9 (0.9)
Leisure	3.3 (0.8)	3.5 (1.1)
Physical safety	4.2 (0.7)	4.2 (1.0)
Energy level	2.6 (1.0)	2.8 (1.1)
Perceived stress scale (0 to 16)	5.9 (3.4)	6.0 (2.9)
*Chronic pain coping inventory – higher scores indicate more frequent use*		
Guarding (0 to 7)	4.0 (2.3)	4.1 (2.2)
Resting (0 to 7)	3.5 (2.3)	3.5 (1.9)
Asking for assistance (0 to 7)	2.6 (2.3)	2.7 (2.3)
Relaxation (0 to 7)	3.2 (1.9)	2.7 (2.3)
Task persistence (0 to 7)	4.5 (1.9)	3.1 (1.8)
Exercise (0 to 7)	4.3 (1.8)	3.8 (1.9)
Seeking social support (0 to 7)	1.9 (2.1)	2.5 (2.4)
Coping self-statements (0 to 7)	4.2 (2.2)	4.3 (2.1)
Emotional support†	53.4 (7.6)	56.1 (7.1)
*Physical activity in past week, median (IQR)*		
Moderate to Vigorous (minutes/week)	120 (30 to 270)	65 (0 to 145)
Light (minutes/week)	150 (60 to 350)	65 (49.5 to 160)
Sedentary (minutes/week)	185 (120 to 420)	310 (120 to 490)
Fear avoidance beliefs – physical activity (0 to 24)	12.5 (4.5)	11.7 (4.3)
Pain catastrophizing scale (0 to 52)	17.4 (11.2)	13.9 (7.6)
Rumination subscale (0 to 16)	6.0 (3.9)	5.0 (3.4)
Magnification subscale (0 to 12)	4.0 (3.0)	3.2 (2.1)
Hopelessness subscale (0 to 24)	7.3 (5.0)	5.7 (3.4)
Treatment expectations for SBSM (6 to 30)	24.9 (3.7)	23.4 (3.9)
Treatment expectations for Medical care (6 to 30)	23.7 (4.8)	21.5 (3.5)

^†^PROMIS measures are standardized and presented as T-scores where the mean for the U.S. population is 50 with a standard deviation of 10

## Abbreviations

BCT: Behavior change technique
BPS: Biopsychosocial
BRLP: Back-related leg pain
COM: Capability, opportunity, motivation
CT: Communication technique
DC: Chiropractor
ER: Emergency room
LBP: Low back pain
NSAID: Non-steroidal anti-inflammatory drug
PT: Physical therapist
QTF: Quebec task force
SBSM: Supported biopsychosocial self-management
SMT: Spinal manipulation therapy
UMN: University of Minnesota
U.S.: United States
